# Being nice, outgoing, curious, organized, and calm—protective or eroded by workplace bullying? Reciprocal effects of personality and bullying, and mechanisms to explain the associations

**DOI:** 10.3389/fpsyg.2025.1740837

**Published:** 2026-01-05

**Authors:** Michael Rosander, Morten Birkeland Nielsen

**Affiliations:** 1 Department of Behavioural Sciences and Learning, Linköping University, Linköping, Sweden; 2 National Institute of Occupational Health, Oslo, Norway; 3 Department of Psychosocial Science, University of Bergen, Bergen, Norway

**Keywords:** big five, emotional distress, interpersonal conflict, neuroticism, workplace harassment

## Abstract

The present study examined the reciprocal associations between personality traits and workplace bullying, and mechanisms underlying these associations. Data were drawn from a large longitudinal probability sample of the Swedish workforce (*N* = 2,024). Workplace bullying was measured using both self-labelling and behavioural experience methods, and personality was assessed through the Big Five traits. Two mechanisms were proposed and tested: an interpersonal conflict mechanism and an emotional distress mechanism. The results showed reciprocal associations between bullying and neuroticism, indicating that personality may both influence exposure to bullying and be shaped by it. Both mechanisms fully mediated the association from neuroticism to subsequent bullying, suggesting that emotional reactivity and conflict involvement explain why individuals high in neuroticism are at greater risk of becoming targets. Emotional distress also fully mediated the effects from exposure to bullying to subsequent increases in neuroticism and decreases in conscientiousness. These findings imply that prolonged exposure to bullying may erode emotional stability and self-regulatory capacity over time. Overall, the study highlights that in cases of exposure to bullying, personality should not be viewed as a fixed risk factor but as a dynamic system interacting with the social environment, where bullying can gradually alter dispositional functioning through emotional and interpersonal processes.

## 1 Introduction

According to the Golden Rule, you should “do unto others as you would have them do unto you.” From a layperson’s point of view, the Golden Rule might be seen as advice on how to avoid becoming a target of systematic harassment from others in the workplace—commonly known as workplace bullying. In this sense, the logic seems straightforward: just be nice to your colleagues and superiors, be outgoing, stay curious, organized, and calm, and others in the workplace will treat you with the same respect and dignity. However, evidence suggests that the Golden Rule may not fully apply to workplace bullying. While some support for an association between how a person tends to act and the risk of being bullied can be found in cross-sectional studies on personality traits and bullying (Nielsen et al., 2017), the effects are typically small and pale in comparison with environmental risk factors for workplace bullying, such as job design, leadership, and stressors (Salin and Hoel, 2020). The few longitudinal studies that have been conducted provide even less support for the idea that personality traits in general increase the risk of being bullied in any substantial way (e.g., Nielsen and Knardahl, 2015; Persson et al., 2016). There have also been suggestions that being exposed to bullying might, in itself, affect individuals so profoundly that it could lead to changes in their personality. Yet, in line with the notion that personality traits are relatively stable patterns of thoughts, feelings, and behaviours that shape how individuals respond across situations (Cervone and Pervin, 2019; Roberts, 2009), there is no strong evidence that exposure to bullying substantially alters a target’s personality (e.g., Farley et al., 2025; Nielsen and Knardahl, 2015). Nevertheless, although relatively stable, traits do not imply rigid or mechanical reactions, but rather patterns of behaviour that are meaningfully consistent while still allowing for contextual variation (Roberts, 2009). There is evidence suggesting that personality can change as a result of adverse or traumatic life events (Bucciol et al., 2024; Löckenhoff et al., 2009). Workplace bullying, being an extreme social stressor (Zapf and Einarsen, 2005) and a threat demand (Tuckey et al., 2015), may constitute such a traumatic event.

Taken together, existing evidence on the relation between workplace bullying and personality traits is limited and inconclusive, and to date we do not know how they are related. That is, although a growing body of research has linked personality traits (e.g., agreeableness, neuroticism, conscientiousness) to experiences of workplace bullying, much of this evidence is based on cross-sectional data, which limits conclusions about directionality and causality (Nielsen et al., 2017). To understand the causal relation between personality characteristics and bullying, longitudinal data are required. However, to date only five studies have used this type of design: three have examined the full Five-Factor Model of personality (Farley et al., 2025; Nielsen and Knardahl, 2015; Podsiadly and Gamian-Wilk, 2017), while two have focused on a subset of traits (Bowling et al., 2010; Persson et al., 2016). Three of these previous studies were relatively large, yet none employed a true probability sample which is a requirement for external validity. With the exception of one smaller study (Podsiadly and Gamian-Wilk, 2017), previous research has also relied solely on a single-item measure of bullying rather than using behavioural experience inventories, which is the recommended approach for assessing this phenomenon (Nielsen et al., 2020). Moreover, no longitudinal study has yet tested potential mechanisms that might explain the associations between personality and bullying. Hence, there are important gaps in our understanding of the relation between personality characteristics and workplace bullying.

To address these gaps in research, this two-part study draws on a large longitudinal national probability sample of Swedish employees, applying both behavioural-experience and self-labelling measures of bullying to determine time-lagged associations between workplace bullying and personality. The first part replicates previous research—yet, as the first large-scale study, it employs a more refined approach to measuring bullying—and examines reciprocal relations between exposure to bullying and the personality traits in the Five-Factor Model. The second part extends previous research by examining involvement in conflicts, perceived stress, and symptoms of mental health problems as mechanisms that may explain significant paths established in the first part of the study.

## 2 Part 1: the relationships between exposure to workplace bullying and personality

Workplace bullying is characterised by prolonged and systematic mistreatment of an employee by others in the workplace (Einarsen et al., 2020b). From the outset, or as a result of the bullying process, a growing power imbalance develops between perpetrator and target. Over time, such treatment results in the target feeling increasingly helpless (Rosander and Nielsen, 2025) and with a reduced ability to respond or put an end to the behaviours. In the European tradition, bullying is typically investigated from the perspective of the victim, as intent on behalf of the perpetrator may easily be rationalized as merely jargon while the consequences for the target are the same no matter the intent (Einarsen et al., 2020b). Workplace bullying is a major and detrimental problem across the world. Estimates indicate that 10–15 percent of the working population may be exposed to varying degrees of bullying (Nielsen et al., 2010). In Sweden, 14 percent are exposed to bullying behaviours at a level where negative outcomes clearly differentiate them from those not exposed, while about 6 percent are more systematically targeted (Rosander and Blomberg, 2019; Rosander et al., 2024). These figures indicate that bullying remains a substantial and persistent problem in the Swedish workforce, seriously affecting the lives of hundreds of thousands of employees in Sweden and millions worldwide. The outcomes of bullying range from work-related issues such as job dissatisfaction (e.g., Steele et al., 2022), sickness absence (e.g., Burr et al., 2022), and expulsion from working life (e.g., Glambek et al., 2015) to health issues such as depression and anxiety (Verkuil et al., 2015), as well as suicidal ideation (Luo et al., 2023) and actual suicides (Conway et al., 2022; Magnusson Hanson et al., 2023), underscoring its severity as a workplace problem. The seriousness and extreme consequences of bullying could contribute to changes in how one perceives the world and relates to others (Janoff-Bulman, 1992). As bullying represents a direct threat to personal integrity, with severe consequences among those exposed, it has been argued to be a potential traumatic event that may even shatter the target’s basic assumptions about the world as benevolent, and therefore also could alter a person at the core, including personality characteristics (e.g., Farley et al., 2025; Nielsen and Knardahl, 2015).

Personality is defined as “psychological systems that contribute to an individual’s enduring and distinctive patterns of experience and behaviour” (Cervone and Pervin, 2019, p. 4); that is, characteristics that influence individual actions, show relative consistency over time, and are distinct enough to differentiate between people. Many different categorizations of personality have been proposed; however, there is now strong consensus and substantial evidence that individual differences can be organised into five broad dimensions (John, 2022), known as the Five-Factor Model: *Agreeableness*, reflecting compassion, cooperation, and a tendency to be considerate toward others; *Conscientiousness*, involving organization, responsibility, and self-discipline in pursuing goals; *Extraversion*, characterized by sociability, enthusiasm, and a preference for stimulation and interaction; *Openness to Experience*, denoting curiosity, imagination, and a willingness to engage with new ideas and experiences; and *Neuroticism*, representing emotional instability, sensitivity to stress, and a tendency to experience negative emotions such as anxiety or moodiness. Although the Big Five framework has been operationalized through several measurement traditions—such as lexical approaches, questionnaire-based models, and observer ratings—they converge strongly on this five-dimensional structure (Goldberg, 1993; McCrae and John, 1992).

It has been suggested that personality can be both an outcome and a precursor of workplace bullying (Zapf and Einarsen, 2020). In the following, we first outline how personality traits may represent a risk factor for subsequent exposure to bullying, and thereafter elaborate on how bullying can lead to changes in personality traits.

### 2.1 Personality as a risk factor for exposure to bullying

Personality as a risk factor for workplace bullying must be considered carefully, taking into account not only victim characteristics but also how perpetrators perceive them and how organizational shortcomings in handling and preventing bullying allow such behaviour to emerge. Victim precipitation theory (Elias, 1986) suggests that certain traits or behaviours may inadvertently trigger mistreatment, though this idea is controversial as it can imply victim blame. Originally, it referred to situations where victims more clearly initiated conflict (Wolfgang, 1957), but later applications emphasize how perceived provocation or vulnerability can shape victimization.

From a social interactionist perspective (Tedeschi and Felson, 1994), personality may influence how individuals are perceived and treated in social contexts. Traits such as high conscientiousness or neuroticism may lead others to view a person as demanding or difficult, thereby increasing the likelihood of negative reactions (Aquino and Thau, 2009; Tepper et al., 2006). In this view, aggression is purposeful behaviour used to enforce social or performance norms. When such reactions are not managed, they can escalate into bullying. Thus, it is not personality itself but how certain traits are *interpreted and responded to* within the workplace that creates risk.

Building on school bullying research (Olweus, 1993), Zapf and Einarsen (2020) distinguish two personality-linked pathways: the *vulnerable victim*, characterised by insecurity and high neuroticism, and the *provocative victim*, marked by irritability, low agreeableness, or aggression. Vulnerable individuals may invite *predatory bullying* (Einarsen, 1999), whereas provocative victims may display behaviours that provoke negative reactions or mutual hostility, potentially setting in motion a cycle of escalating conflict (Baillien et al., 2016)—that is, *dispute-related bullying* (Einarsen, 1999). Taken together, personality contributes to the risk of bullying through its social meaning rather than inherent traits. When personal characteristics are misinterpreted or not managed properly in organizational contexts characterized by, for example, weak leadership and poor conflict management, they may become catalysts for persistent mistreatment.

### 2.2 Bullying as a precursor to changes in personality

Although personality is regarded as a relatively stable property of adults (Roberts, 2009), it may change through self-regulatory processes across the lifespan (Hennecke et al., 2014). Such changes may arise from experiences of discrepancies between one’s internal state and external standards, originating, for example, from social or environmental conditions (McCrae and Costa, 2008), including adverse or traumatic life events. Theoretically, exposure to traumatic events may shatter basic assumptions about the world as benevolent and meaningful (Janoff-Bulman, 1992), leading to enduring changes in how individuals relate to others. Regarding the Five-Factor Model, traumatic experiences have been particularly associated with increased neuroticism and decreased agreeableness and conscientiousness (Bucciol et al., 2024; Löckenhoff et al., 2009). Hence, it seems reasonable to assume that exposure to workplace bullying could lead to changes in personality traits, either temporary or more permanent.

While traumatic events represent one possible pathway to change, the TESSERA framework (Triggering situations, Expectancy, States/State expressions, and ReActions; Wrzus and Roberts, 2017) highlights another: personality development as the outcome of repeated sequences of everyday experiences. Building on state–trait research within a lifespan perspective (e.g., Fleeson, 2001; Roberts et al., 2008), the framework proposes that each sequence—beginning with a situational trigger and followed by expectations, state expressions, and reactions—contributes incrementally to long-term trait change when repeated over time. As workplace bullying represents a form of repeated and prolonged exposure, it can be seen as a context in which such negative sequences are frequently activated: recurrent exposure to mistreatment creates expectations of hostility, evokes states of anxiety or defensiveness, and leads to reactions of withdrawal or conflict. When accumulated over time, these repeated sequences may gradually shift an individual’s personality profile—for instance, increasing neuroticism or reducing extraversion (Farley et al., 2025).

### 2.3 Previous research on personality and workplace bullying

Summarizing cross-sectional studies by means of meta-analysis, Nielsen et al. (2017) found that exposure to workplace bullying was significantly associated with extraversion, neuroticism, agreeableness, and conscientiousness, but not openness to experience. Bullying was most strongly associated with neuroticism across the included studies. While there does not appear to be a clear and consistent victim profile (Glasø et al., 2007), there are indications that targets may differ from those not exposed, showing higher scores on neuroticism and lower agreeableness and conscientiousness (Glasø et al., 2009; Matthiesen and Einarsen, 2001). However, these studies do not clarify whether specific personality traits increase the likelihood of being singled out as a target, or whether the differences in trait levels among those bullied are the result of exposure to bullying—that is, whether they should be regarded as outcomes of the bullying process or as pre-existing characteristics constituting risk factors for bullying. This distinction is central to understanding whether personality functions primarily as a cause or a consequence of bullying, and shedding light on the direction of the association requires longitudinal evidence.

However, to date, longitudinal evidence remains scarce, and the findings are inconclusive. For example, when examining all Five-Factor Model traits, Nielsen and Knardahl (2015) found support for neuroticism as an antecedent of bullying, but when controlling for role conflict and role ambiguity—both well-established risk factors for bullying (den Van Brande et al., 2016)—the association disappeared, leaving only conscientiousness as a predictor of bullying across the two-year study period. In the same study, bullying predicted changes in agreeableness, conscientiousness, and openness over time. Persson et al. (2016) examined only neuroticism and extraversion and found that becoming bullied increased neuroticism, while no longer being bullied decreased neuroticism and increased extraversion. In a small study within a single private corporation, Podsiadly and Gamian-Wilk (2017) found that exposure to bullying behaviours predicted a decrease in agreeableness over time. In a large-scale four-year study, Farley et al. (2025) investigated personality change as an outcome of exposure to bullying and found that exposure was related to increased neuroticism and decreased extraversion, and that changes in bullying status were also associated with decreased conscientiousness. There are studies that have focused on specific aspects of personality traits in relation to the risk of bullying. Reknes et al. (2021) found that trait anxiety and trait anger affected the development of bullying over a six-month period. Zahlquist et al. (2022) investigated the same traits as potential links between conflict involvement and concurrent exposure to bullying behaviours over a 30-day period. They found that only trait anger moderated the association—that is, high conflict involvement combined with high trait anger was associated with a higher level of exposure to bullying behaviours.

### 2.4 Research questions

As findings diverge across studies, the reciprocal effects between personality and bullying remain uncertain. Moreover, previous research has employed different methodological strategies and operationalised bullying in diverse ways. Hence, there is a need for further large-scale studies that employ longitudinal designs and consistent methods for measuring bullying (Nielsen and Knardahl, 2015). In response to this call, the present study addresses the following research questions using time-lagged data from a large national probability sample of Swedish employees.

*Research Question 1*: How is baseline personality, as defined by the Five-Factor Model, associated with subsequent changes in exposure to workplace bullying, and how is baseline exposure to bullying associated with subsequent changes in personality traits?

*Research Question 2*: Do the cross-lagged associations between personality and bullying differ depending on whether bullying is measured using self-labelling or behavioural experience?

In the second part of the study, we build on the longitudinal associations identified in Part 1 by examining potential mechanisms that may account for these relations.

## 3 Methods (part 1)

The study is based on a longitudinal probability sample of the entire Swedish working population, excluding individuals under 18 years of age and those employed at workplaces with fewer than ten employees. Statistics Sweden (a governmental agency) was responsible for sampling and data collection. Baseline data were collected over a two-month period in the autumn of 2024, and follow-up data were collected in the spring of 2025, beginning 7 months after the initial wave. A total of 2,024 individuals responded at both time points. For each wave, information about the survey—including all details necessary for informed consent—was distributed digitally to all individuals with an active digital mailbox, and by post to those without. In each wave, three reminders were issued to non-respondents, the second of which was sent by post to all who had not yet replied. Once data collection was complete, Statistics Sweden appended information from the Swedish National Population Register to the dataset, including variables such as biological sex, age, education, and foreign-born status. Access to the anonymized dataset was granted only after this step, which strengthened the study’s ethical integrity. All procedures were carried out in accordance with the principles of the Declaration of Helsinki and applicable national ethical guidelines. The study was approved by the Swedish Ethical Review Authority (Protocol No. 2023-06603-01).

### 3.1 Participants

The sample consisted of 58% women and 42% men, with a mean age of 49.44 years (SD = 10.50). On average, participants had worked at their current workplace for 10.11 years (SD = 9.62), and 96% held a permanent position. A clear majority (89%) were born in Sweden. In terms of educational attainment, 16% had completed compulsory education, 33% had graduated from upper secondary school, and 51% held a university degree.

### 3.2 Measures

Workplace bullying was assessed using the Negative Acts Questionnaire–Revised (NAQ–R; Einarsen et al., 2009), validated for use in a Swedish context (Rosander et al., 2024). The instrument includes 22 items describing negative and unwanted behaviours that employees may experience at work. When such acts occur repeatedly and systematically over time, they may constitute bullying. Responses were recorded on a five-point frequency scale ranging from *never* to *daily*. Internal consistency was high (Cronbach’s ⍺ = 0.89 and McDonald’s *ω* = 0.91 at both T1 and T2). At T1, those who reported being exposed to at least some bullying behaviours were asked how long the exposure had been ongoing, with a six-point frequency scale ranging from *recently* to *more than 2 years*.

Self-labelled bullying was also assessed. First, respondents were presented with the following definition: “Bullying occurs when a person, repeatedly and over time, is subjected to negative treatment by one or more individuals, in situations where the person has difficulty defending themselves. A conflict between two equally strong individuals is not considered bullying.” They were then asked: “How often have you been subjected to bullying at your workplace during the past 6 months?” Responses were given on the same five-point frequency scale as used for the NAQ–R.

Personality was measured using the 20-item version of the International Personality Item Pool (IPIP; Goldberg, 1999), known as the Mini-IPIP (Donnellan et al., 2006). This instrument is based on the Five-Factor Model of personality and includes four items for each of the five traits: agreeableness (*α* = 0.75/0.76; *ω* = 0.76/0.76), conscientiousness (*α* = 0.72/0.74; *ω* = 0.76/0.72), extraversion (*α* = 0.80/0.82; *ω* = 0.81/0.82), openness (*α* = 0.67/0.68; *ω* = 0.78/0.70), and neuroticism (*α* = 0.76/0.78; *ω* = 0.74/0.77). Participants were asked, “How well do the following statements apply to you?,” and responses were given on a seven-point Likert scale (1–7).

Sex, age, education, and foreign-born status, drawn directly from the Swedish National Population Register, were included as covariates in the main analyses, as all have been associated with the occurrence of workplace bullying (Einarsen et al., 2020a). Their inclusion is also important because the stability of personality traits has been shown to differ across demographic groups (Löckenhoff et al., 2008).

### 3.3 Data analyses

All statistical analyses were conducted using Stata version 19.5. Internal consistency was assessed using Cronbach’s alpha (*α*) and McDonald’s omega (*ω*). Alpha is reported to facilitate comparability with previous studies, whereas omega provides a complementary model-based estimate of reliability. Omega coefficients were computed using the omegacoef module (Shaw, 2020) in Stata. To investigate the reciprocal relationships between personality and workplace bullying over time, a two-wave cross-lagged panel model was estimated. The model included autoregressive paths for each construct from Time 1 to Time 2, as well as cross-lagged paths from each of the five personality traits at T1 to bullying at T2, and from bullying at T1 to each personality trait at T2. All variables were modelled as observed variables, and the analysis controlled for sex, age, education, and foreign-born status. Structural equation modelling (SEM) was applied using maximum likelihood estimation with missing values (MLMV). Model fit was assessed using the chi-square statistic (χ^2^), root mean square error of approximation (RMSEA), comparative fit index (CFI), and Tucker–Lewis index (TLI). RMSEA values below 0.05, and CFI and TLI values close to 0.95, were considered indicative of good fit (Hu and Bentler, 1998). Because workplace bullying is a highly skewed variable (as most individuals are not exposed), all coefficients were estimated using 5,000 bootstrap samples.

## 4 Results (part 1)

To investigate Research Question 1, a cross-lagged model was tested, including paths from bullying measured using behavioural experience (NAQ–R) at T1 to all five personality traits at T2, as well as from each trait at T1 to bullying at T2. The model showed good fit to the data, 𝜒^2^(20) = 83.16, *p* < 0.001, CFI = 0.995, TLI = 0.981, RMSEA = 0.040, 95% CI [0.031, 0.048]. The addition of the cross-lagged paths significantly improved model fit compared to a stability model including only autoregressive paths, 𝜒^2^(10) = 25.24, *p* = 0.005.

**Figure 1:**
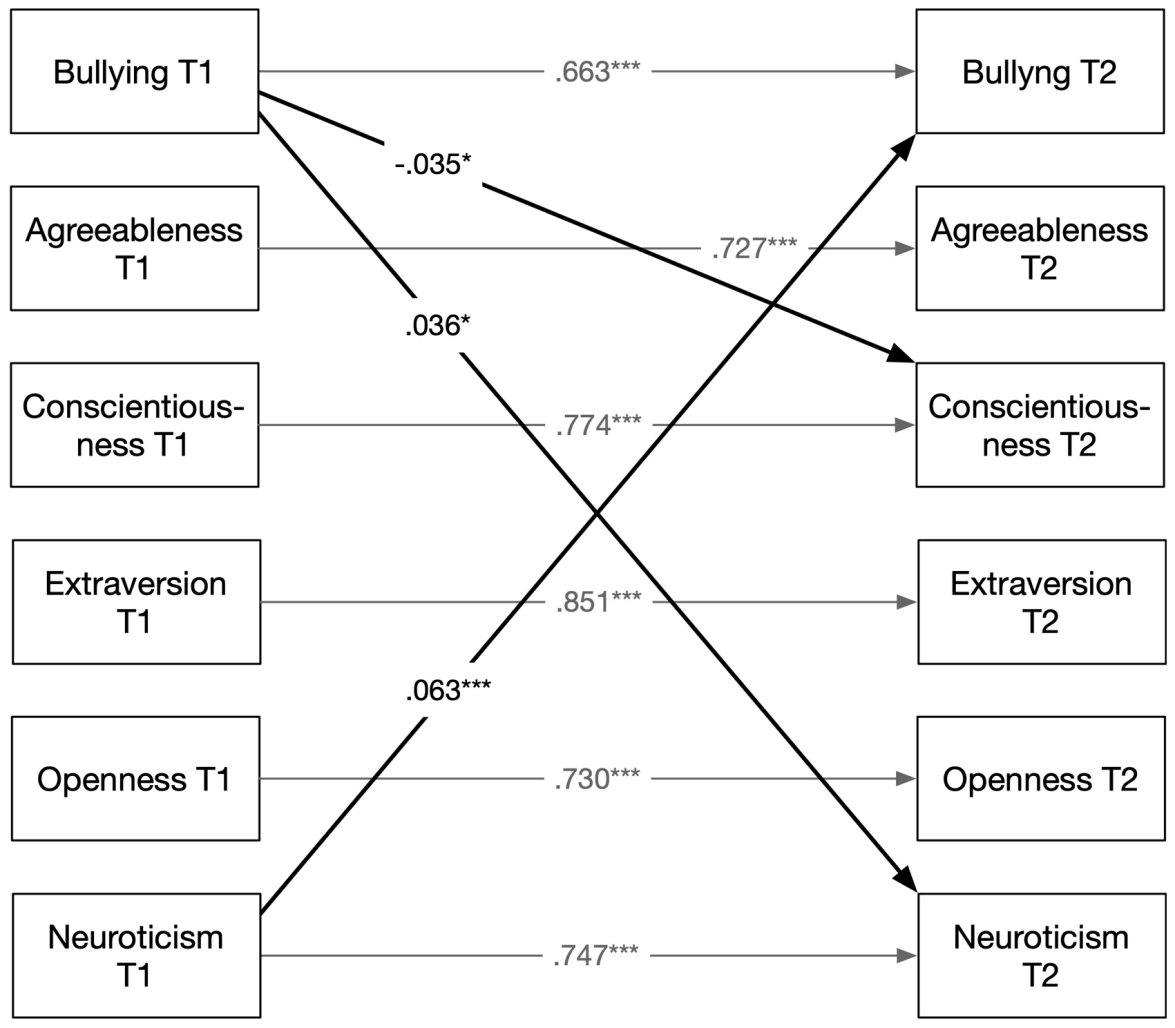
Cross-lagged effects. Standardized coefficients based on 5,000 bootstrap samples. Bullying, Negative Acts Questionnaire–Revised. Covariates: sex, age, education, and foreign-born status. *p < 0.05. ***p < 0.001.

In Figure 1, only the significant cross-lagged paths are presented (all coefficients are presented in Supplementary Table S2). In the direction from bullying to personality, conscientiousness (*β* = −0.035, *p* = 0.041) and neuroticism (*β* = 0.036, *p* = 0.019) at T2 were significantly predicted. In the reverse direction, neuroticism at T1 significantly predicted bullying at T2 (*β* = 0.063, *p* = 0.002). It should be noted that the majority of those exposed to bullying behaviours at T1 reported that the mistreatment had been ongoing for some time. Only 17% indicated that it had begun recently, whereas 57% reported being exposed for at least a year, with half of them reporting exposure for more than 2 years.

Table 1 presents means, standard deviations, and intercorrelations between the covariates and the variables involved in the significant paths of the model (full descriptive results are provided in Supplementary Table S1). Bullying, neuroticism, and conscientiousness were all significantly associated with the background variables, thereby also providing empirical support for their inclusion as covariates. Furthermore, as expected, there are significant zero-order correlations between the personality variables and bullying. Notably, the association was stronger for neuroticism compared to conscientiousness. For both traits, the magnitude of the correlation was similar across both the concurrent and longitudinal measures, reflecting the temporal stability demonstrated in the main analysis.

**Table 1:** Means, standard deviations, and intercorrelations for the significant cross-lagged paths in the model and the covariates.

Variable	Mean	SD	1	2	3	4	5	6	7	8
1. Sex	0.58	0.49	–							
2. Age	49.44	10.50	0.01^ns^	–						
3. Education	4.04	1.24	0.10	−0.22	–					
4. Foreign-born	0.11	0.31	−0.003^ns^	−0.02^ns^	0.03^ns^	–				
5. Bullying T1	1.22	0.30	0.10	−0.08	−0.02^ns^	0.06**	–			
6. Bullying T2	1.21	0.30	0.09	−0.10	−0.01^ns^	0.04^ns^	0.68	–		
7. Neuro T1	3.22	1.09	0.18	−0.20	0.06**	0.02^ns^	0.30	0.27	–	
8. Neuro T2	3.21	1.10	0.17	−0.18	0.06**	0.01^ns^	0.27	0.32	0.77	–
9. Consc T2	5.37	0.98	0.07**	0.13	−0.11	0.06**	−0.09	−0.10	−0.25	−0.32

Sex: women = 1, men = 0. Foreign-born: 1 = born abroad, 0 = born in Sweden. Neuro, Neuroticism; Consc, Conscientiousness; Bullying, Negative Acts Questionnaire–Revised.

All correlations significant at *p* < 0.001 except where indicated: **p < 0.01. ns, not significant.

In regard to Research Question 2, the same model, but with bullying now measured using self-labelling, was tested. Also this model showed good fit to the data, 𝜒^2^(20) = 85.42, *p* < 0.001, CFI = 0.994, TLI = 0.978, RMSEA = 0.040, 95% CI [0.032, 0.049]. The only significant cross-lagged path was from baseline neuroticism to bullying at follow-up, *β* = 0.083, *p* < 0.001. All coefficients are presented in Supplementary Table S3.

## 5 Summary (part 1)

The results indicated that participants’ personality were relatively stable over time; however, there was evidence of change in levels of conscientiousness and neuroticism at follow-up among respondents reporting higher exposure to workplace bullying. Nonetheless, the cross-lagged effects were small, which is expected when stability paths are included in the model. In the reverse direction—baseline personality predicting subsequent changes in exposure to bullying—only neuroticism emerged as a significant predictor. Although this effect was also modest, it was stronger than the cross-lagged effects from bullying to personality.

Interestingly, neuroticism was the only trait to predict exposure regardless of how bullying was measured. When self-labelled bullying was used instead of the behavioural-experience inventory, the previously observed effects from bullying at baseline to later changes in conscientiousness and neuroticism were no longer significant. Previous research has identified neuroticism as an antecedent of workplace bullying (Nielsen and Knardahl, 2015; Persson et al., 2016), although only the latter study reported a change in neuroticism following exposure. Notably, both studies used self-labelling as the operationalization of bullying. With time-lagged associations between personality traits and bullying now established, the next critical question concerns the mechanisms underlying these associations. This question will be addressed in Part 2 of the study.

## 6 Part 2: explaining reciprocal associations between exposure to bullying and personality traits

In Part 2 of this study, we address the three significant cross-lagged paths established in Part 1 (presented in Figure 1). We explore mechanisms that might help explain these associations. First, we outline the theoretical rationale for the proposed underlying mechanisms and then specify the hypotheses to be tested.

### 6.1 Theoretical rationale for underlying mechanisms

Previous studies have provided indications of reciprocal associations between workplace bullying and personality (e.g., Farley et al., 2025; Nielsen and Knardahl, 2015). However, the underlying psychological processes that might explain why personality contributes to bullying exposure, and why bullying in turn may shape personality, remain poorly understood. In the following sections, we present a theoretical rationale for these links, outlining why we expect (a) involvement in conflicts, (b) perceived stress, and (c) symptoms of mental health problems to be three key mechanisms. Specifically, based on the results from Part 1, we examine these factors as potential mechanisms through which neuroticism may act as a risk factor for bullying and through which bullying may influence subsequent change in neuroticism and conscientiousness.

### 6.2 Neuroticism as a predictor of subsequent bullying

*Neuroticism* reflects individual differences in emotional stability versus emotional reactivity (Cervone and Pervin, 2019). It indicates whether a person generally feels calm, secure, and relaxed, or tends to be anxious, tense, and easily upset. Individuals high on neuroticism are more likely to experience emotions such as anxiety, anger, irritability, guilt, and depression. Explicitly displaying such emotions could, according to victim precipitation theory (Elias, 1986), act as a trigger for harassment, as such reactions may be perceived by others as norm violations—both in terms of social conduct and performance expectations (Tedeschi and Felson, 1994). In the context of bullying, a provocative victim may elicit frustration in others and thereby provoke aggressive behaviour or engage in interactions that escalate conflict (Zapf and Einarsen, 2020). That is, as employees scoring high on neuroticism tend to be anxious and easily upset, their behaviour may be perceived by others as more provocative than that of their more emotionally stable colleagues. Hence, neurotic employees may face an increased risk of being bullied because they behave in ways that elicit aggression from others (Nielsen and Knardahl, 2015). According to this line of reasoning, high levels of neuroticism may activate an *interpersonal conflict mechanism* that mediates the link between personality and exposure to bullying.

In terms of a vulnerable victim (Zapf and Einarsen, 2020), a disposition to express negative affect—encompassing emotions such as anxiety, worry, sadness, and depression—may also elicit mistreatment. In contrast to the provocative victim, the vulnerable victim may signal weakness, being less likely to retaliate if attacked and instead tending to withdraw. They may be perceived as demanding or interpersonally difficult, and thus seen as triggering aggression, but could also simply represent the most convenient outlet for frustration in cases of predatory bullying (Einarsen, 1999). This type of personality trait has shown the most consistent links to various indicators of victimization (Aquino and Thau, 2009). This suggests that depression, anxiety, and perceived stress may function as an *emotional distress mechanism* mediating the association between neuroticism and exposure to bullying.

However, the emotional distress mechanism could also be understood as a tendency to perceive behaviours as more negative, in line with the gloomy perception hypothesis (de Lange et al., 2005)—that is, the victim perceive others’ behaviours as more negative and more personally directed. In this sense, individuals high in neuroticism may interpret ambiguous social interactions as hostile or rejecting, thereby perceiving greater exposure to bullying behaviours even in the absence of actual increases in mistreatment.

Taken together, we suggest that the association between neuroticism and subsequent bullying can be understood through two distinct mechanisms: an *interpersonal conflict mechanism* (conflict involvement) and an *emotional distress mechanism* (perceived stress, and symptoms of depression and anxiety).

*Hypothesis 1*: Neuroticism at baseline has an indirect effect on subsequent exposure to workplace bullying via (a) involvement in conflicts and (b) perceived stress and symptoms of mental health problems.

### 6.3 Change in personality following exposure to bullying

Exposure to workplace bullying represents a severe and prolonged social stressor (Zapf and Einarsen, 2005) that may have enduring consequences for personality development. Repeated experiences of devaluation, exclusion, and loss of control can elicit persistent stress and negative affect, which, over time, may become internalised as stable patterns of emotional reactivity. According to the reformulated learned helplessness model (Abramson et al., 1978), such experiences promote feelings of helplessness and hopelessness by fostering stable and global attributions of failure and lack of agency. These emotional states can develop into enduring dispositions of worry, tension, and vulnerability to stress—consistent with a more neurotic personality profile.

From a broader trauma perspective, exposure to bullying can also be understood in terms of shattered assumptions (Janoff-Bulman, 1992). Persistent humiliation and social exclusion challenge fundamental beliefs about the world as benevolent and meaningful and about the self as competent and valued. When these assumptions are violated, individuals may become chronically vigilant to threat and rejection, further reinforcing negative affectivity and emotional instability. Thus, stress, anxiety, and depressive reactions may not only represent temporary strain but also form the emotional distress mechanism through which bullying contributes to heightened neuroticism over time.

Taken together, these perspectives suggest that exposure to bullying may foster increases in neuroticism through the *emotional distress mechanism*, whereby stress, anxiety, and depressive reactions become internalised as more enduring patterns of emotional instability.

*Hypothesis 2*: Exposure to workplace bullying at baseline has an indirect effect on changes in neuroticism at follow-up via perceived stress and symptoms of mental health problems.

At the same time, bullying may undermine the self-regulatory resources required for persistence, organization, and control. Conservation of resources theory (Hobfoll, 1989) posits that chronic stress and loss of valued resources trigger spirals of depletion, in which reduced energy and motivation impair goal-directed behaviour. Through the same emotional distress mechanism, prolonged exposure to stress and mental health symptoms may erode conscientiousness—manifesting in disengagement, avoidance, and reduced self-discipline. Consequently, bullying-related stress reactions may operate as a common pathway linking exposure to decreased conscientiousness.

Accordingly, the emotional distress mechanism may also account for reductions in conscientiousness, as prolonged stress and mental health symptoms undermine self-regulation, persistence, and goal-directed behaviour.

*Hypothesis 3*: Exposure to workplace bullying at baseline has an indirect effect on changes in conscientiousness at follow-up via perceived stress and symptoms of mental health problems.

## 7 Methods (part 2)

Part 2 of this study employs the same sample, participants, statistical software, and procedures as Part 1.

### 7.1 Measurements

In addition to the instruments used in Part 1, Part 2 includes the following inventories:

Symptoms of anxiety and depression were measured using the Hospital Anxiety and Depression Scale (HADS; Zigmond and Snaith, 1983). The instrument includes 14 items—seven assessing anxiety symptoms and seven assessing depression symptoms—referring to experiences during the past week. Each item is rated on a four-point scale (0–3). An example anxiety item is “I feel tense or ‘wound up’,” with response options ranging from not at all to most of the time. A corresponding example for depression is “I have lost interest in my appearance.” Internal consistency was *α* = 0.87/0.87; *ω* = 0.85/0.86 for anxiety and *α* = 0.84/0.85; *ω* = 0.88/0.88 for depression.

Conflict involvement was measured using a single item:” How often have you experienced difficulties cooperating, tensions, or been in conflict with one or more people at your workplace during the past 6 months?” Responses were given on a five-point frequency scale from 0 = *never* to 4 = *very often*.

Perceived stress was also measured using a single item:” Have you felt stressed at work during the past month?” Responses were given on a five-point frequency scale from 0 = *not at all* to 4 = a*lmost all the time*.

### 7.2 Data analysis

**Figure 2:**
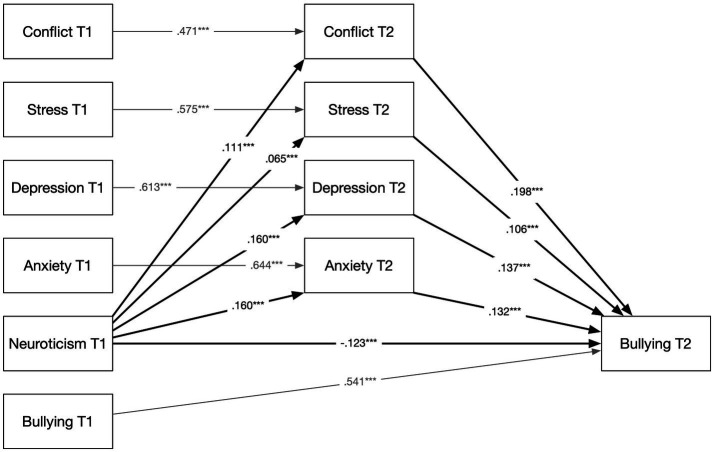
Mediation model: neuroticism at T1 predicting bullying at T2 via conflict involvement and emotional distress. Standardized coefficients based on 5,000 bootstrap samples. Covariates: sex, age, education, and foreign-born status. ***p < 0.001.

**Figure 3:**
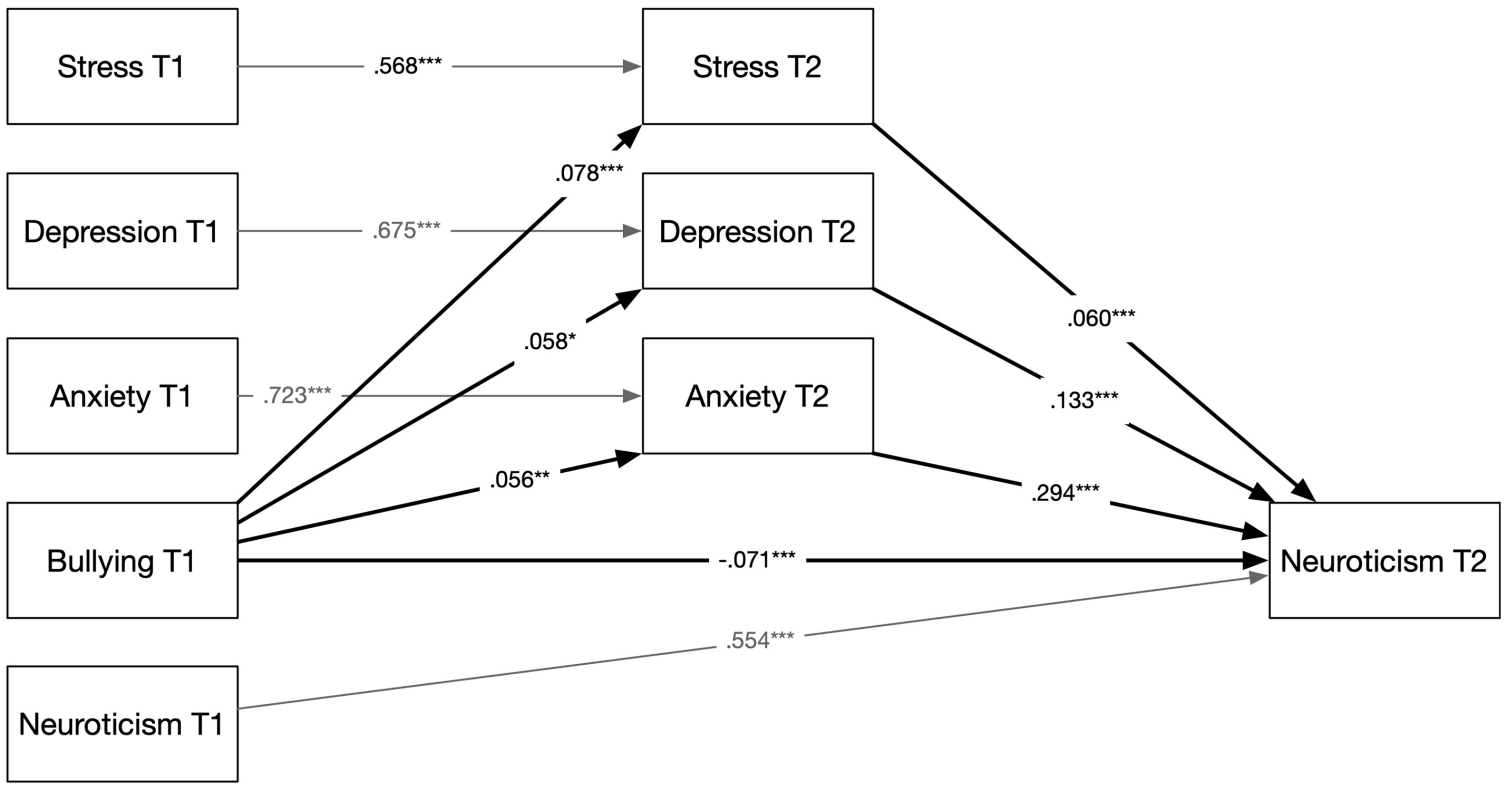
Mediation model: bullying at T1 predicting neuroticism at T2 via emotional distress. Standardized coefficients based on 5,000 bootstrap samples. Covariates: sex, age, education, and foreign-born status. *p < 0.05. **p < 0.01. ***p < 0.001.

**Figure 4:**
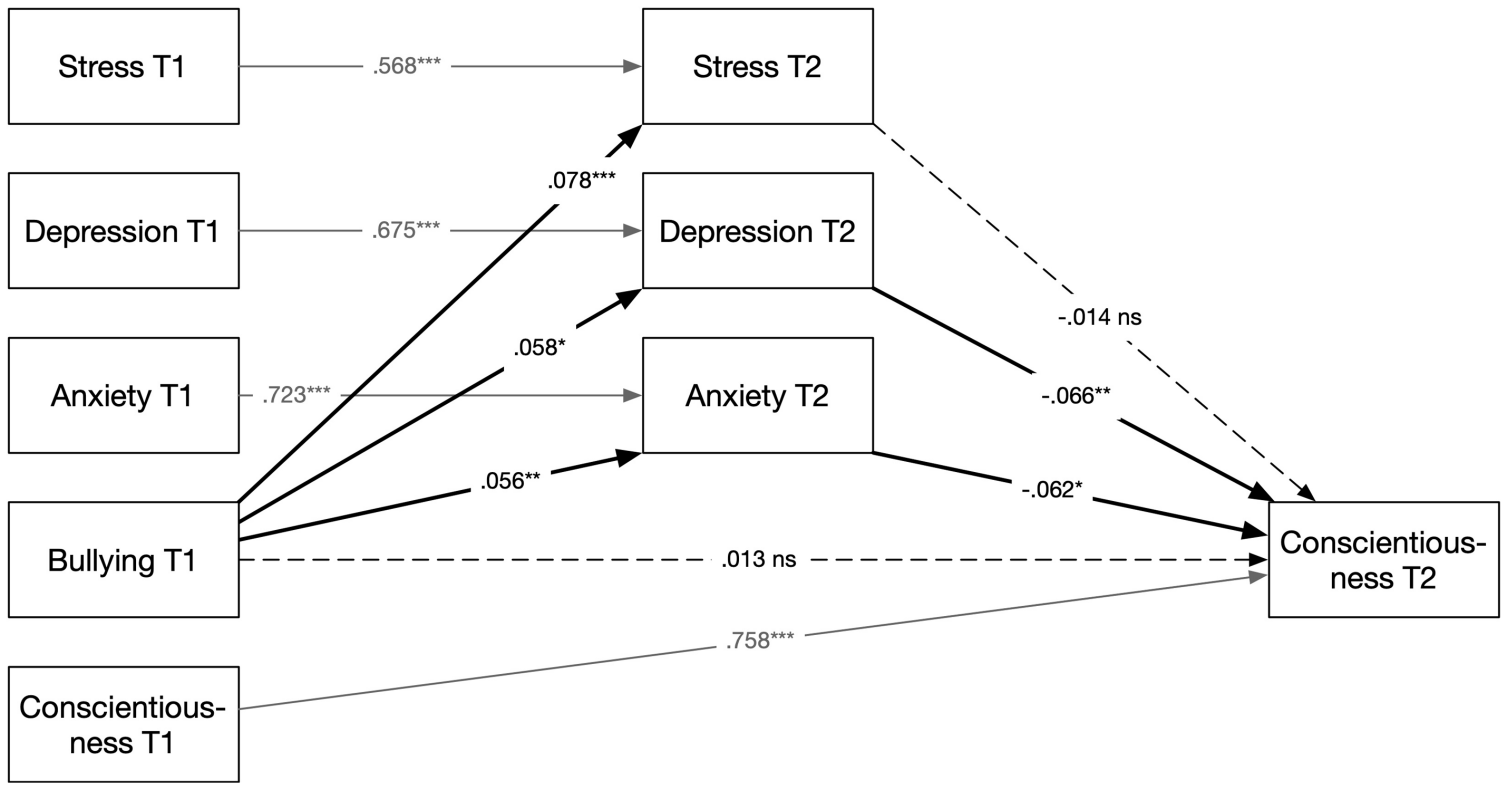
Mediation model: bullying at T1 predicting conscientiousness at T2 via emotional distress. Standardized coefficients based on 5,000 bootstrap samples. Covariates: sex, age, education, and foreign-born status. *p < 0.05. **p < 0.01. ***p < 0.001. ns, not significant.

Based on the significant associations established in Part 1, follow-up mediation models that included baseline measures for all mediators and outcomes were tested. Indirect effects were estimated using 5,000 bootstrap samples, which is the recommended approach for testing mediation because the product of the *a* and *b* paths is not normally distributed and bootstrap confidence intervals therefore provide a more accurate basis for inference (Hayes, 2022). Baron and Kenny’s (1986) stepwise procedure, which for a long time served as a common framework for testing mediation, has since been shown to be overly restrictive, and the bootstrap test is almost always more powerful than the Sobel test they recommended (Zhao et al., 2010). In contemporary mediation analysis, the presence of a significant indirect effect is sufficient to establish mediation (Hayes, 2022). All covariates were the same as in the cross-lagged model. Results are presented graphically in Figures 2–4; however, all results, including 95% bootstrapped confidence intervals (BootCI), are also presented in Supplementary Tables S4–S6.

## 8 Results (part 2)

Table 2 presents means, standard deviations, and intercorrelations for the variables included in the mediation analyses. As expected, there were significant zero-order correlations between the predictors (neuroticism and exposure to bullying at T1), the proposed mediators (conflict involvement, perceived stress, depression, and anxiety symptoms at T2), and the outcomes (bullying at T2, neuroticism at T2, and conscientiousness at T2). The weakest correlations were observed for conscientiousness. Notably, neuroticism showed strong associations with both anxiety and depression symptoms, consistent with prior research. These patterns support the theoretical rationale for testing the proposed mediation effects.

**Table 2:** Means, standard deviations, and intercorrelations between main variables in the mediation analyses.

Variable	Mean	SD	1	2	3	4	5	6	7	8
1. Bullying T1	1.22	0.30	–							
2. Bullying T2	1.21	0.30	0.68	–						
3. Conflict T2	1.00	0.92	0.39	0.48	–					
4. Stress T2	1.90	1.18	0.30	0.40	0.31	–				
5. Depression T2	0.47	0.47	0.35	0.44	0.31	0.38	–			
6. Anxiety T2	0.73	0.60	0.38	0.47	0.35	0.51	0.71	–		
7. Neuro T1	3.22	1.09	0.30	0.28	0.28	0.30	0.50	0.60	–	
8. Neuro T2	3.21	1.10	0.27	0.32	0.32	0.40	0.59	0.70	0.77	–
9. Consc T2	5.37	0.98	−0.09	−0.10	−0.11	−0.14	−0.27	−0.30	−0.25	−0.32

Neuro, Neuroticism; Consc, Conscientiousness; Bullying, Negative Acts Questionnaire–Revised.

All correlations significant at *p* < 0.001.

To test Hypothesis 1, a mediation analysis was conducted (see Figure 2). Controlling for sex, age, education, and foreign-born status, as well as baseline levels of all mediators and the outcome, the results showed a significant indirect effect of neuroticism at T1 on bullying at T2 [*β* = 0.018, *p* < 0.001, 95% BootCI (0.014, 0.023)]. The analysis supported both the interpersonal conflict mechanism [via conflict involvement, *β* = 0.006, *p* < 0.001, 95% BootCI (0.003, 0.081)] and the emotional distress mechanism [via stress, depression, and anxiety, *β* = 0.013, *p* < 0.001, 95% BootCI (0.009, 0.017)]. Thus, Hypothesis 1 was supported. The association between baseline neuroticism and subsequent exposure to bullying was fully mediated by conflict involvement, perceived stress, anxiety, and depression. When these variables were included as mediators, the direct path from neuroticism to bullying became negative [*β* = −0.123, *p* < 0.001, 95% BootCI (−0.162, −0.085)], suggesting a suppression effect. That is, it is not neuroticism per se that increases the risk of bullying, but rather the aspects of neuroticism that manifest through conflict involvement and emotional distress. Once this shared variance was accounted for, the remaining aspects of neuroticism were not associated with increased bullying risk and may even show a weak protective tendency.

Testing Hypothesis 2, a similar mediation analysis was conducted (see Figure 3). In this model, only the emotional distress mechanism (via stress, depression, and anxiety) was included. Using the same control variables and accounting for baseline levels of all mediators and the outcome, the results showed a significant indirect effect of exposure to bullying at T1 on neuroticism at T2 [*β* = 0.103, *p* = 0.002, 95% BootCI (0.038, 0.168)]. Hypothesis 2 was supported. As in the previous model, the direct effect of baseline bullying exposure on subsequent neuroticism was fully mediated, with the direct path turning negative [*β* = −0.071, *p* < 0.001, 95% BootCI (−0.100, −0.042)]—suggesting a suppression effect.

Finally, testing Hypothesis 3, a corresponding mediation model predicting conscientiousness at T2 was conducted (see Figure 4). The same control variables as in the previous model together with baseline levels of the mediators and the outcome were adjusted for. The results showed a significant indirect effect of exposure to bullying at T1 on conscientiousness at T2 [*β* = −0.028, *p* = 0.007, 95% BootCI (−0.047, −0.007)]. Hypothesis 3 was supported. The direct path from bullying to conscientiousness was not significant [*β* = 0.0131, *p* = 0.511, 95% BootCI (−0.025, 0.051)], showing that any association operates through the emotional distress mechanism rather than a direct effect of bullying on conscientiousness.

## 9 Discussion

The aims of this study were to examine the reciprocal associations between exposure to workplace bullying and personality traits (Part 1) and to test two mechanisms proposed to explain the significant associations identified in the first part of the study—an *interpersonal conflict mechanism* and an *emotional distress mechanism* (Part 2). Neuroticism was reciprocally associated with bullying, while exposure to bullying was also associated with changes in conscientiousness. The association between baseline neuroticism and subsequent bullying was fully mediated by both mechanisms, while the associations between baseline exposure to bullying and subsequent neuroticism and conscientiousness were fully mediated by the emotional distress mechanism.

Although research in this area is still limited, previous studies on the Big Five model (Farley et al., 2025; Nielsen and Knardahl, 2015), as well as the present study suggest that neuroticism is the trait most consistently associated with bullying—both as a risk factor and as an outcome. As a risk factor, individuals high in neuroticism may display behaviours that evoke frustration in others at the workplace. The results showed that neuroticism predicted subsequent bullying through both the emotional distress and the interpersonal conflict mechanisms, each of which may influence how individuals interact with others at work. From a social interactionist perspective (Tedeschi and Felson, 1994), behaviours that are interpreted as violations of social or performance norms may trigger attempts by others to correct the behaviour and restore it to what is considered acceptable. When such reactions are poorly managed, they may manifest as bullying behaviours.

That neuroticism predicted subsequent bullying does not necessarily imply that personality in itself constitutes a risk factor. Trait activation theory (Tett and Burnett, 2003) suggests that personality traits are expressed when situational cues activate them. From this perspective, a stressful and conflict-prone work environment may activate traits associated with neuroticism, which in turn elicits stronger emotional reactions and higher levels of conflict involvement, thereby increasing the risk of becoming targeted. Thus, rather than neuroticism per se being a direct cause of bullying, it may be the interaction between individual dispositions and adverse work environments that explains why certain traits appear to increase vulnerability.

The work environment plays a crucial role in shaping the risk of bullying (Einarsen et al., 1994). Nielsen and Knardahl (2015) found that the association between neuroticism and later bullying disappeared when adjusting for two key indicators of a poor psychosocial work environment—role conflict and role ambiguity. This aligns with the notion that individual risk factors, such as mental health problems, no longer increase vulnerability when the work environment is sufficiently sound (Rosander, 2021). In a well-functioning workplace, employees with mental health problems do not face a heightened risk of being exposed to bullying. Moreover, when other well-known risk factors such as role stress (Salin and Hoel, 2020) are reduced and the conflict management climate is strong (Blomberg et al., 2025; Einarsen et al., 2018), the likelihood that frustration will escalate into interpersonal conflict or coercive actions aimed at correcting perceived misfitting behaviours—and eventually into bullying—is substantially diminished. This line of reasoning challenges the assumptions of victim precipitation theory (Elias, 1986), however, the effects of personality, the mechanisms identified in the present study, and their interaction with the work environment warrant further research to better understand how personality influences exposure to bullying.

The results also indicated that exposure to bullying may affect neuroticism and conscientiousness over time. Personality traits are not considered entirely rigid and may change—most notably during adolescence, but also across the lifespan (Hennecke et al., 2014; Roberts, 2009). This was also reflected in the present study, with significant correlations between age and both neuroticism and conscientiousness (see Table 1). The results showed an increase in neuroticism with greater exposure to bullying behaviours. Based on the notion of bullying as an extreme stressor and a threat demand (Tuckey et al., 2015; Zapf and Einarsen, 2005), such exposure may undermine a person’s basic trust in and beliefs about others (Janoff-Bulman, 1992), thereby eroding the target’s fundamental sense of security. This is likely to apply most strongly to those who have only recently begun to experience bullying. However, the results also showed that most participants had been exposed for a longer period, meaning that the experience was no longer a sudden shock for them. According to the TESSERA framework (Wrzus and Roberts, 2017), changes in personality traits can also result from repeated sequences of exposure—an aspect that lies at the core of how workplace bullying is defined—which could help explain the link.

We also found that the association with increased neuroticism could be understood through the emotional distress mechanism. This means that the effect of bullying is not merely a temporary strain; rather, stress, anxiety, and depressive reactions resulting from exposure—although initially possibly transient—may, when repeated or prolonged, have a more profound impact on the target, potentially developing into an enduring disposition. Repeated and systematic exposure to negative behaviours may lead to learned helplessness, fostering global attributions of hopelessness that manifest as stronger neurotic tendencies (Abramson et al., 1978; Rosander and Nielsen, 2025).

The results indicated a reciprocal relationship between exposure to bullying and neuroticism, suggesting the risk of a vicious circle in which exposure leads to emotional distress that over time may result in changes in the person’s disposition. This altered disposition may in turn affect interpersonal relationships and generate further emotional distress. A similar vicious cycle has been proposed for bullying and psychological distress (Nielsen et al., 2012).

An interesting additional finding concerned a potential suppression effect for neuroticism, observed both when it was tested as an antecedent and as an outcome of bullying. In the model where neuroticism predicted subsequent exposure to bullying, the direct path became negative when mediators were included. This suggests that it is not neuroticism per se that increases the risk of bullying, but rather the emotional and interpersonal correlates of neuroticism—manifested through stress, anxiety and depression symptoms, and conflict involvement—that account for the observed association. Once these reactive components were statistically controlled for, the remaining aspects of neuroticism (such as emotional awareness, sensitivity, or cautiousness) may even serve a protective function, reducing the likelihood of being targeted. In this sense, the suppression effect highlights that the risk lies not in the trait itself, but in how its affective expressions interact with the social environment.

A similar suppression effect emerged when bullying was tested as a predictor of later neuroticism. When emotional distress variables were included in the model, the direct path from bullying to neuroticism became negative, suggesting that the apparent increase in neuroticism was fully explained by stress, and anxiety and depressive symptoms. Once these transient emotional states were accounted for, the remaining variance in neuroticism may instead reflect adaptive emotional regulation or coping attempts in response to prolonged stress. Hence, the suppression effect in this direction implies that while bullying initially heightens emotional reactivity, some individuals may later develop compensatory mechanisms that dampen or mask further increases in neuroticism.

The results also showed a change in conscientiousness as an outcome of exposure to bullying. This change was fully mediated through the emotional distress mechanism, indicating that bullying increases stress as well as anxiety and depressive symptoms, which in turn may develop into a more enduring disposition. From a conservation of resources perspective (Hobfoll, 1989), this implies that bullying may undermine the self-regulatory resources required for persistence, organization, and control. As an extreme and prolonged stressor, bullying may deplete valued personal resources and trigger spirals of depletion that gradually erode goal-directedness and self-regulatory behaviour—both central aspects of conscientiousness (John, 2022). In this case, the direct effect was not significant, suggesting that the association between bullying and reduced conscientiousness was fully mediated by emotional distress. This process may also be linked to feelings of helplessness that bullying can elicit (Rosander and Nielsen, 2025), reflecting diminished agency and control in the face of sustained adversity.

Another notable finding was that when using self-labelled bullying instead of behavioural experience as the outcome, the cross-lagged associations from bullying at baseline to subsequent changes in personality traits disappeared. One plausible explanation is that self-labelling reflects a subjective interpretation of social experiences, in which individuals already frame themselves as victims of bullying. This self-appraisal may stabilise how individuals relate to their environment, thereby reducing the likelihood of further personality change. In contrast, the behavioural experience measure captures variation in exposure to negative acts more objectively and continuously, which may make it more sensitive to detecting gradual personality shifts over time.

Despite this difference, the association from neuroticism to subsequent bullying remained significant regardless of measurement method. This suggests that individuals high in neuroticism may be generally more vulnerable to negative interpersonal experiences at work. High neuroticism is associated with heightened sensitivity to everyday stressors and a tendency toward negative interpretation (Suls and Martin, 2005), which may explain why such individuals are more prone to perceiving themselves as targets of bullying, irrespective of how it is measured. They are also more likely to perceive ambiguous social cues as hostile or threatening—a pattern consistent with the concept of hostile attribution bias (Epps and Kendall, 1995). This interpretation is further supported by the gloomy perception hypothesis (de Lange et al., 2005). That is, the consistent association between neuroticism and workplace bullying—across both behavioural experience and self-labelled measures—may indicate that individuals high in neuroticism are inclined to view ambiguous or unfavourable interpersonal experiences in a negative light. This heightened sensitivity may increase their likelihood of perceiving mistreatment, whether in terms of reporting frequent negative acts or identifying themselves as victims of bullying.

### 9.1 Practical implications

The present results have several practical implications for how organizations can prevent and respond to workplace bullying. First, the finding of a reciprocal relationship between bullying and neuroticism points to a potential vicious circle in which exposure to bullying increases emotional distress, which in turn could alter dispositions in ways that heighten vulnerability to further mistreatment. This underscores the need for early intervention to break this cycle—by addressing emotional strain and conflict involvement before they consolidate into more enduring patterns of reactivity and relational difficulties.

Second, because emotional distress emerged as the central mechanism explaining all significant associations, attention should be directed towards the emotional consequences of bullying rather than assumed personality weaknesses. Providing timely access to psychological support and opportunities for recovery may prevent short-term stress and anxiety from developing into lasting dispositions of emotional instability or reduced self-regulation.

Third, the finding that conscientiousness decreased indirectly through emotional distress highlights that bullying not only undermines well-being but may also erode goal-directedness, organization, and self-discipline. Restoring a sense of control and agency among targets—through supportive leadership, clear work structures, and manageable demands—may therefore be essential for recovery and reintegration.

Finally, these findings suggest that preventive efforts should target the interaction between individual dispositions and the work environment rather than viewing personality as a fixed risk factor. Strengthening the conflict management climate, ensuring fair procedures, and reducing stressors such as role conflict and ambiguity can limit the activation of traits that otherwise make some individuals more vulnerable in adverse environments. By focusing on both the emotional and organizational mechanisms identified here, organizations can more effectively mitigate the personal and collective costs of workplace bullying.

### 9.2 Strengths and limitations

A notable strength of the study is the large longitudinal probability sample of the Swedish workforce. One could argue that the time lag is too short to detect changes in personality resulting from exposure to bullying. This is not necessarily a limitation, but it requires discussion. Few, if any, participants started to experience bullying at the same time as the baseline survey was distributed—most had already been exposed to bullying behaviours for some time. The majority had been exposed for at least a year at T1, and about one third of those for more than 2 years. Thus, the timeframe of the study is in reality longer than merely the period between T1 and T2, which has important implications for the interpretation of the results. Because all outcomes and mediators at T2 were adjusted for their T1 levels, the cross-lagged and indirect paths reflect incremental change from T1 to T2. Given that most bullied participants had been exposed well before T1, any change in personality traits that occurred prior to baseline is not captured. This implies that the estimates presented here are *conservative* with respect to the cumulative impact of bullying. Nevertheless, different timeframes should be examined in future research to provide a more detailed understanding of how the bullying process interacts with personality traits.

A further strength of the study is that it was the first to investigate the associations between personality traits and workplace bullying using two separate methods: self-labelling and behavioural experience. This provided a more nuanced picture of how bullying and personality traits are connected. Moreover, in all analyses, we adjusted for T1 levels of all mediators and outcomes, thereby modelling change rather than static levels. Controlling for prior levels typically absorbs a substantial portion of variance through stability paths, which often reduces the strength of other effects. This also contributed to *conservative* estimates and should be regarded as a notable strength. Furthermore, because most individuals are not exposed to workplace bullying, the phenomenon is highly skewed; therefore, all models were estimated using 5,000 bootstrapped samples—a method that reduces the impact of deviations from normality.

The data are based on self-reports, which could have affected the results, as there is a possibility of subjective interpretations, social desirability, and common method variance (Spector, 2006). However, the main variables of the study are of such a nature that anything other than subjective interpretation would probably introduce more error, as the participants’ subjective experience plays a crucial role in understanding the outcomes. For this reason, alternative and potentially more objective measures might not be appropriate (Nielsen et al., 2020). If social desirability influenced the responses, this would most likely contribute to underestimations of the effects, as exposure to bullying—particularly self-labelled bullying—is less likely to be reported from a social desirability standpoint. Finally, the longitudinal design and the presentation of the central variables in different sections of the questionnaire are factors that may have reduced the likelihood of common method variance (Podsakoff et al., 2003).

Although the Mini-IPIP is a well-validated instrument (Donnellan et al., 2006), it provides a more abbreviated assessment of the Big Five traits than other personality measures that capture these dimensions more comprehensively. This means that some nuances within each trait may not have been detected. The analyses were also based on observed scores, which may introduce measurement error. However, given the longitudinal survey design and the focus on change over time, this approach represents a reasonable and pragmatic choice. Future research could examine whether similar patterns emerge when using more extensive personality measures and latent-variable modelling.

Part of how we have discussed the findings is based on how bullies might react to individuals displaying different personality traits. We do not have data on the reasoning or perceptions of the bullies—we only have data from potential targets. Whether bullies actually perceive certain targets as provoking or otherwise triggering their negative behaviour therefore remains unknown. This is something that future studies should explore further.

Taken together, these strengths and limitations suggest that the results provide a conservative yet robust indication of how personality and bullying interact over time, while pointing to areas where future longitudinal designs could further refine this understanding.

### 9.3 Conclusion

The present study provides new insights into the complex interplay between personality and workplace bullying. The findings indicate reciprocal associations between bullying and neuroticism, showing that personality may influence exposure to bullying while also being shaped by it. As a novel contribution, we introduced two mechanisms to explain these associations—the interpersonal conflict mechanism and the emotional distress mechanism. Together, the two mechanisms fully mediated the effect of neuroticism on subsequent bullying, suggesting that neuroticism may contribute to behaviours or interactions that escalate tension and conflict at work, and that heightened emotional reactivity further increases vulnerability to bullying.

Changes in personality traits as outcomes of exposure to bullying behaviours were, in addition to neuroticism, also found for conscientiousness, and for both traits the emotional distress mechanism fully mediated the association. For conscientiousness, this implies that prolonged exposure to bullying can undermine goal-directedness and self-regulatory capacity.

Overall, the findings highlight that personality should not be regarded as a fixed risk factor but as a dynamic system that interacts with the social environment. Bullying can gradually erode emotional stability and conscientiousness, but these effects occur through emotional and interpersonal processes rather than through the traits themselves. Preventing and addressing bullying is therefore not only a matter of protecting well-being but also of maintaining the psychological resources that sustain adaptive personality functioning over time.

## Data Availability

The raw data supporting the conclusions of this article will be made available by the authors, without undue reservation.
