# Supplementary material for: Being nice, outgoing, curious, organized, and calm—protective or eroded by workplace bullying? Reciprocal effects of personality and bullying, and mechanisms to explain the associations
Source: Front Psychol. 2026 Jan 5;16:1740837. doi: 10.3389/fpsyg.2025.1740837 (PMC12812535; doi:10.3389/fpsyg.2025.1740837)
Supplement: Supplementary file 1 [file Supplementary_Material.pdf]

Supplementary Material

Being Nice, Outgoing, Curious, Organized, and Calm—Protective or Eroded by Workplace Bullying? Reciprocal Effects of Personality and Bullying, and Mechanisms to Explain the Associations

Michael Rosander and Morten Birkeland Nielsen

Table S1

Means, standard deviations, and intercorrelations for all variables in the cross-lagged models and the covariates

Variable	Mean	SD	1.	2.	3.	4.	5.	6.	7.	8.	9.	10.	11.	12.	13.	14.	15.	16.	17.
1. Sex	0.58	0.49	–																
2. Age	49.44	10.50	.01^{ns}	–															
3. Education	4.04	1.24	.10	-.22	–														
4. Foreign-born	0.11	0.31	-.03^{ns}	-.02^{ns}	.03^{ns}	–													
5. NAQ–R T1	1.22	0.30	.10	-.08	-.02^{ns}	.06**	–												
6. NAQ–R T2	1.21	0.30	.09	-.10	.01^{ns}	.04^{ns}	.68	–											
7. SLB T1	1.08	0.36	.08	.00^{ns}	-.03^{ns}	.07**	.57	.39	–										
8. SLB T2	1.08	0.35	.08	.01^{ns}	-.00^{ns}	.01^{ns}	.36	.53	.40	–									
9. Agree T1	5.56	0.89	.25	.04^{ns}	.10	-.07**	-.02^{ns}	-.02^{ns}	.07**	.05*	–								
10. Agree T2	5.56	0.90	.26	.06**	.12	-.07**	-.03^{ns}	-.03^{ns}	.03^{ns}	.04^{ns}	.76	–							
11. Consc T1	5.43	0.94	.08	.10	-.12	.05*	-.07**	-.06**	.02^{ns}	-.00^{ns}	.17	.12	–						
12. Consc T2	5.37	0.98	.07**	.13	-.11	.06**	-.09	-.10	-.01^{ns}	.02^{ns}	.15	.18	.79	–					
13. Extra T1	4.23	1.17	.00^{ns}	.07**	-.01^{ns}	-.03^{ns}	-.06**	-.06**	.02^{ns}	-.04^{ns}	.33	.30	.15	.14	–				
14. Extra T2	4.23	1.20	.01^{ns}	.08	.00^{ns}	-.02^{ns}	-.04*	-.07**	.02^{ns}	-.02^{ns}	.31	.37	.12	.16	.86	–			
15. Open T1	5.26	1.00	-.05*	-.04^{ns}	.19	.01^{ns}	-.02^{ns}	.00^{ns}	.01^{ns}	.01^{ns}	.33	.28	.06**	.02^{ns}	.26	.25	–		
16. Open T2	5.28	1.00	-.05*	-.06**	.21	-.02^{ns}	-.03^{ns}	-.02^{ns}	-.01^{ns}	-.02^{ns}	.27	.38	.03^{ns}	.07**	.26	.31	.75	–	
17. Neuro T1	3.22	1.09	.18	-.12	.06**	.02^{ns}	.30	.27	.13	.13	-.12	-.09	-.29	-.25	-.24	-.21	-.10	-.08	–
18. Neuro T2	3.21	1.10	.17	-.12	.06**	.01^{ns}	.27	.32	.11	.15	-.12	-.15	-.28	-.32	-.23	-.26	-.08	-.13	.77

Note. Sex: women = 1, men = 0. Foreign-born: 1 = born abroad, 0 = born in Sweden. Agree = Agreeableness, Consc = Conscientiousness, Extra = Extraversion, Open = Openness to Experience, Neuro = Neuroticism. Bullying = Negative Acts Questionnaire–Revised. SLB = Self-Labelled Bullying. All correlations significant at $p < .001$ except where indicated: $^{*}p < .05$. $^{**}p < .01$. ^{ns} = not significant.

Supplementary Material

Table S2

Cross-lagged model (Research Question 1). Bullying measured by the NAQ–R

Variable	b	Boot SE	95% BootCI	p
**Agreeableness T2**				
Bullying T1	-0.037	0.049	[-0.133, 0.059]	.448
Agreeableness T1	0.726	0.017	[0.696, 0.757]	< .001
Sex	0.134	0.028	[0.080, 0.188]	< .001
Age	0.003	0.001	[0.001, 0.006]	.011
Education	0.038	0.011	[0.016, 0.060]	.001
Foreign-born status	-0.067	0.048	[-0.161, 0.026]	.159
**Conscientiousness T2**				
Bullying T1	***-0.114***	***0.055***	***[-0.225, -0.008]***	***.041***
Conscientiousness T1	0.800	0.014	[0.772, 0.828]	< .001
Sex	0.022	0.027	[-0.032, 0.075]	.419
Age	0.004	0.001	[0.002, 0.007]	.001
Education	-0.012	0.011	[-0.033, 0.009]	.250
Foreign-born status	0.070	0.049	[-0.025, 0.166]	.158
**Extraversion T2**				
Bullying T1	0.028	0.055	[-0.077, 0.138]	.615
Extraversion T1	0.852	0.011	[0.831, 0.873]	< .001
Sex	0.019	0.028	[-0.036, 0.073]	.492
Age	0.003	0.001	[-0.000, 0.005]	.065
Education	0.013	0.011	[-0.009, 0.036]	.238
Foreign-born status	0.027	0.049	[-0.069, 0.121]	.579
**Openness T2**				
Bullying T1	-0.056	0.054	[-0.162, 0.049]	.296
Openness T1	0.724	0.015	[0.694, 0.755]	< .001
Sex	-0.047	0.029	[-0.105, 0.010]	.111
Age	-0.002	0.001	[-0.004, 0.001]	.274
Education	0.059	0.013	[0.034, 0.084]	< .001
Foreign-born status	-0.087	0.057	[-0.201, 0.022]	.123
**Neuroticism T2**				
Bullying T1	***0.131***	***0.056***	***[0.020, 0.241]***	***.019***
Neuroticism T1	0.745	0.015	[0.715, 0.776]	< .001
Sex	0.067	0.032	[0.002, 0.130]	.040
Age	-0.002	0.002	[-0.005, 0.001]	.147
Education	0.006	0.013	[-0.020, 0.032]	.640
Foreign-born status	-0.022	0.053	[-0.129, 0.078]	.669
**Bullying T2**				
Agreeableness T1	0.003	0.006	[-0.010, 0.015]	.683
Conscientiousness T1	0.004	0.006	[-0.008, 0.016]	.466
Extraversion T1	-0.001	0.004	[-0.010, 0.008]	.812
Openness T1	0.004	0.005	[-0.005, 0.014]	.385
Neuroticism T1	***0.017***	***0.006***	***[0.006, 0.028]***	***.002***
Bullying T1	0.659	0.042	[0.580, 0.746]	< .001
Sex	0.008	0.010	[-0.011, 0.028]	.406
Age	-0.001	0.001	[-0.002, 0.000]	.154
Education	0.001	0.004	[-0.007 0.009]	.822
Foreign-born status	-0.001	0.017	[-0.032, 0.034]	.976

Supplementary Material

Table S3

Cross-lagged model (Research Question 2). Bullying measured as self-labelled victimization

Variable	b	Boot SE	95% BootCI	p
**Agreeableness T2**				
Victimization T1	-0.056	0.038	[-0.138, 0.014]	.132
Agreeableness T1	0.728	0.015	[0.698, 0.757]	< .001
Sex	0.135	0.028	[0.081, 0.189]	< .001
Age	0.003	0.001	[0.001, 0.006]	.009
Education	0.037	0.011	[0.015, 0.059]	.001
Foreign-born status	-0.065	0.048	[-0.156, 0.028]	.172
**Conscientiousness T2**				
Victimization T1	-0.068	0.046	[-0.165, 0.018]	.138
Conscientiousness T1	0.803	0.015	[0.774, 0.832]	< .001
Sex	0.019	0.027	[-0.035, 0.072]	.489
Age	0.005	0.001	[0.002, 0.007]	< .001
Education	-0.012	0.010	[-0.033, 0.008]	.264
Foreign-born status	0.067	0.049	[-0.027, 0.163]	.167
**Extraversion T2**				
Victimization T1	0.002	0.043	[-0.079, 0.088]	.960
Extraversion T1	0.852	0.011	[0.830, 0.874]	< .001
Sex	0.020	0.028	[-0.034, 0.075]	.459
Age	0.002	0.001	[-0.000, 0.005]	.066
Education	0.013	0.011	[-0.009, 0.035]	.248
Foreign-born status	0.028	0.050	[-0.069, 0.122]	.574
**Openness T2**				
Victimization T1	-0.030	0.045	[-0.122, 0.054]	.505
Openness T1	0.724	0.015	[0.694, 0.754]	< .001
Sex	-0.048	0.030	[-0.107, 0.010]	.104
Age	-0.001	0.001	[-0.004, 0.001]	.315
Education	0.059	-0.000	[0.033, 0.083]	< .001
Foreign-born status	-0.088	0.056	[-0.200, 0.020]	.113
**Neuroticism T2**				
Victimization T1	0.043	0.048	[-0.050, 0.139]	.373
Neuroticism T1	0.753	0.015	[0.723, 0.782]	< .001
Sex	0.069	0.033	[0.004, 0.134]	.035
Age	-0.002	0.001	[-0.005, 0.001]	.117
Education	0.005	0.013	[-0.020, 0.032]	.696
Foreign-born status	-0.017	0.052	[-0.120, 0.084]	.744
**Victimization T2**				
Agreeableness T1	0.012	0.009	[-0.008, 0.029]	.223
Conscientiousness T1	0.007	0.009	[-0.012, 0.025]	.448
Extraversion T1	-0.011	0.008	[-0.026, 0.004]	.146
Openness T1	0.004	0.000	[-0.010, 0.020]	.556
Neuroticism T1	***0.026***	***0.009***	***[0.009, 0.044]***	***.001***
Victimization T1	0.375	0.059	[0.270, 0.496]	< .001
Sex	0.017	0.014	[-0.010, 0.044]	.217
Age	0.001	0.001	[-0.001, 0.002]	.256
Education	0.001	0.006	[-0.010, 0.013]	.796
Foreign-born status	-0.018	0.020	[-0.059, 0.021]	.351

Supplementary Material

Table S4

Mediation model (Hypothesis 1).

Variable	b	Boot SE	95% BootCI	p
**→ Conflict T2**				
Neuroticism T1 →	0.093	0.018	[0.057, 0.129]	< .001
Conflict T1 →	0.475	0.024	[0.427, 0.523]	< .001
Sex	0.072	0.036	[0.003, 0.143]	.045
Age	-0.005	0.002	[-0.008, -0.001]	.007
Education	-0.011	0.014	[-0.039, 0.017]	.445
Foreign-born status	-0.014	0.058	[-0.125, 0.100]	.804
**→ Stress T2**				
Neuroticism T1 →	0.070	0.022	[0.028, 0.113]	.001
Stress T1 →	0.561	0.019	[0.524, 0.598]	< .001
Sex	0.092	0.042	[0.010, 0.174]	.028
Age	-0.004	0.002	[-0.008, 0.000]	.058
Education	0.048	0.017	[0.015, 0.082]	.004
Foreign-born status	-0.110	0.067	[-0.237, 0.023]	.104
**→ Depression T2**				
Neuroticism T1 →	0.068	0.009	[0.051, 0.086]	< .001
Depression T1 →	0.612	0.024	[0.565, 0.658]	< .001
Sex	-0.024	0.015	[-0.054, 0.006]	.116
Age	-0.001	0.001	[-0.002, 0.000]	.108
Education	-0.006	0.006	[-0.018, 0.006]	.299
Foreign-born status	0.000	0.025	[-0.050, 0.049]	.997
**→ Anxiety T2**				
Neuroticism T1 →	0.087	0.011	[0.064, 0.110]	< .001
Anxiety T1 →	0.642	0.022	[0.599, 0.685]	< .001
Sex	-0.012	0.017	[-0.045, 0.022]	.493
Age	-0.003	0.001	[-0.004, -0.001]	.001
Education	0.012	0.007	[-0.000, 0.026]	.059
Foreign-born status	-0.036	0.028	[-0.089, 0.018]	.196
**→ Bullying T2**				
Conflict T2 →	0.061	0.007	[0.047, 0.074]	< .001
Stress T2 →	0.026	0.004	[0.017, 0.034]	< .001
Depression T2 →	0.083	0.017	[0.049, 0.116]	< .001
Anxiety T2 →	0.062	0.014	[0.036, 0.091]	< .001
Neuroticism T1 →	-0.032	0.005	[-0.042, -0.022]	< .001
Bullying T1 →	0.516	0.039	[0.444, 0.598]	< .001
Sex	0.012	0.008	[-0.004, 0.028]	.164
Age	0.000	0.000	[-0.001, 0.001]	.617
Education	0.001	0.004	[-0.006, 0.008]	.832
Foreign-born status	0.006	0.015	[-0.020, 0.039]	.576

Supplementary Material

Table S5

Mediation model (Hypothesis 2).

Variable	b	Boot SE	95% BootCI	p
**→ Stress T2**				
Bullying T1 →	0.310	0.092	[0.131, 0.499]	< .001
Stress T1 →	0.554	0.019	[0.517, 0.592]	< .001
Sex	0.103	0.041	[0.021, 0.183]	.013
Age	-0.005	0.002	[-0.009, -0.001]	.018
Education	0.051	0.017	[0.019, 0.084]	.003
Foreign-born status	-0.122	0.068	[-0.255, 0.011]	.072
**→ Depression T2**				
Bullying T1 →	0.091	0.039	[-0.019, 0.172]	.017
Depression T1 →	0.674	0.021	[0.631, 0.715]	< .001
Sex	-0.002	0.015	[-0.030, 0.026]	.872
Age	-0.002	0.001	[-0.003, -0.001]	.007
Education	-0.004	0.006	[-0.016, 0.008]	.545
Foreign-born status	-0.005	0.026	[-0.054, 0.047]	.857
**→ Anxiety T2**				
Bullying T1 →	0.114	0.042	[0.033, 0.197]	.006
Anxiety T1 →	0.721	0.018	[0.686, 0.755]	< .001
Sex	0.004	0.017	[-0.029, 0.038]	.810
Age	-0.004	0.001	[-0.005, -0.002]	< .001
Education	0.013	0.007	[0.000, 0.027]	.050
Foreign-born status	-0.040	0.028	[-0.095, 0.014]	.156
**→ Neuroticism T2**				
Stress T2 →	0.054	0.014	[0.026, 0.080]	< .001
Depression T2 →	0.300	0.044	[0.215, 0.386]	< .001
Anxiety T2 →	0.517	0.039	[0.441, 0.592]	< .001
Bullying T1 →	-0.252	0.053	[-0.358, -0.151]	< .001
Neuroticism T1 →	0.533	0.017	[0.499, 0.567]	< .001
Sex	0.093	0.028	[0.038, 0.148]	.001
Age	0.001	0.001	[-0.002, 0.004]	.450
Education	-0.002	0.012	[-0.025, 0.020]	.846
Foreign-born status	0.003	0.044	[-0.085, 0.087]	.950

Supplementary Material

Table S6

Mediation model (Hypothesis 3).

Variable	b	Boot SE	95% BootCI	p
**→ Stress T2**				
Bullying T1 →	0.310	0.093	[0.129, 0.495]	.001
Stress T1 →	0.554	0.020	[0.516, 0.593]	< .001
Sex	0.103	0.042	[0.021, 0.186]	.014
Age	-0.005	0.002	[-0.009, -0.001]	.019
Education	0.051	0.017	[0.018, 0.083]	.002
Foreign-born status	-0.122	0.069	[-0.259, 0.013]	.076
**→ Depression T2**				
Bullying T1 →	0.091	0.038	[0.019, 0.171]	.015
Depression T1 →	0.674	0.021	[0.633, 0.714]	< .001
Sex	-0.002	0.015	[-0.031, 0.026]	.874
Age	-0.002	0.001	[-0.003, -0.001]	.007
Education	-0.004	0.006	[-0.016, 0.009]	.541
Foreign-born status	-0.005	0.025	[-0.052, 0.045]	.853
**→ Anxiety T2**				
Bullying T1 →	0.114	0.042	[0.034, 0.200]	.005
Anxiety T1 →	0.721	0.018	[0.684, 0.755]	< .001
Sex	0.004	0.017	[-0.030, 0.038]	.810
Age	-0.004	0.001	[-0.005, -0.002]	< .001
Education	0.013	0.007	[-0.000, 0.027]	.052
Foreign-born status	-0.040	0.028	[-0.093, 0.014]	.150
**→ Conscientiousness T2**				
Stress T2 →	-0.011	0.014	[-0.039, 0.014]	.408
Depression T2 →	-0.137	0.047	[-0.227, -0.043]	.004
Anxiety T2 →	-0.101	0.042	[-0.185, -0.018]	.018
Bullying T1 →	0.042	0.064	[-0.082, 0.170]	.511
Conscientiousness T1 →	0.783	0.015	[0.753, 0.813]	< .001
Sex	0.032	0.027	[-0.020, 0.086]	.235
Age	0.003	0.001	[0.000, 0.005]	.043
Education	-0.013	0.010	[-0.034, 0.008]	.220
Foreign-born status	0.071	0.047	[-0.024, 0.163]	.133
